# David D. Derse, 1949-2009

**DOI:** 10.1186/1742-4690-6-110

**Published:** 2009-12-01

**Authors:** Maureen Shuh

**Affiliations:** 1 Division of Basic Pharmaceutical Sciences, College of Pharmacy, Xavier University of Louisiana, New Orleans, LA 70125, USA

## Abstract

David D. Derse, Ph.D., Head of the Retrovirus Gene Expression Section in the HIV Drug Resistance Program at the National Cancer Institute-Frederick (NCI-Frederick), passed away on October 9, 2009, a scant six weeks after being diagnosed with liver cancer. It was with great sadness that family, friends, and colleagues gathered together for his memorial service on Saturday, October 17, 2009, at the Middletown United Methodist Church in Maryland. As a NCI scientist since 1986, Dave studied the molecular mechanisms of infection and replication of a number of different types of retroviruses. Dave became an internationally known expert on human T cell lymphotrophic viruses type 1 and 2 (HTLV-1 and HTLV-2) and served on the editorial boards of *Virology *and *Retrovirology*. His most recent studies focused on the mechanisms of HTLV-1 virion morphogenesis, transmission, and replication.

## Background

David Daniel Derse was born in Los Angeles, California, on December 22, 1949. After graduating from California State University Northridge with a B.S. in Chemistry in 1973, Dave worked as a research technician at Childrens Hospital Los Angeles from 1974-1977 in the laboratory of Dr. Richard L. Momparler, studying the biochemical pharmacology of new anti-neoplastic agents. Dave earned his Ph.D. in Pharmacology in 1982 from the State University of New York (SUNY) at Buffalo. His graduate adviser at SUNY Buffalo was Dr. Yung-Chi "Tommy" Cheng who moved to the University of North Carolina at Chapel Hill in 1979, and Dave completed his doctoral studies while working with Tommy at UNC. While a graduate student in 1981, Dave co-authored a paper with Dr. Gertrude B. Elion who was later awarded The Nobel Prize in Physiology or Medicine [1]. He trained with Dr. James Casey as a post-doctoral fellow from 1982-1986, first at Louisiana State University Health Sciences Center at New Orleans and later at the National Cancer Institute in Frederick, Maryland. While in the Casey laboratory, Dave identified the enhancer elements in the long terminal repeat that regulate bovine leukemia virus (BLV) gene expression, publishing the data in *Science *[2]. Dave joined NCI-Frederick as a Senior Staff Fellow in 1986, becoming a tenured Senior Investigator in 1991. In 2004, Dave became Head of the Retrovirus Gene Expression Section in the HIV Drug Resistance Program at NCI-Frederick. Dave was also an Adjunct Professor in the graduate program in Genetics at George Washington University and served on the Executive Committee of the Center of Excellence in HIV/AIDS and Cancer Virology, Center for Cancer Research at NCI-Frederick.

During his tenure at NCI-Frederick, Dave identified and characterized the molecular mechanisms of infection, replication, and pathogenesis of different retroviruses, including equine infectious anemia virus (EIAV), bovine leukemia virus (BLV), Moloney murine leukemia virus (MLV), human immunodeficiency virus (HIV), and human T lymphotrophic virus types 1 and 2 (HTLV-1, HTLV-2). In recent years, the focus of Dave's research has been HTLV-1. This work resulted in fundamental insights into both the initial stages of infection and the assembly and release of virions from infected cells (reviewed in [3]). In studies directly comparing the infectivity of HTLV-1 and other retroviral cores, he reported that HTLV-1 is dramatically less efficient that other retroviruses, indicating a post-entry block in replication. He identified motifs critical for the late stage of HTLV-1 assembly and characterized their relative roles in particle release. In 2007, he published studies showing that a peptide motif in the C terminus of the HTLV-1 nucleocapsid (NC) inhibits APOBEC3G (hA3G) packaging into nascent virions, thereby allowing HTLV-1 to evade an important aspect of the body's antiviral defenses [4].

**Figure 1:**
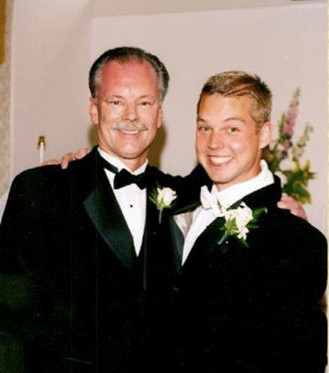
**David and James Derse at the wedding of James and Carrie Derse on July 15, 2000, in Middletown, Maryland**. Reprinted with permission from James Derse.

While Dave is known professionally for his scientific accomplishments, he was perhaps best known by those close to him as a devoted father, grandfather, and uncle. Colleagues appreciated Dave's dry sense of humor, his patience, and his love of teaching. In his personal life, these qualities meant that children were especially drawn to him. From birth to adulthood, the males in his extended family knew Dave as a father figure. Their loss reverberates across three generations. With his son, nephews, and grandsons, he was always actively engaged, whether it was throwing a ball, arm wrestling, fishing, examining stones or seashells close up, planting a tree, experimenting with dry ice, or stretching their imaginations with heroic stories. To both his son James (Figure 1) and his nephew Daniel, one challenged by learning disabilities and the other by deafness, he gave the precious gift of not lowering his expectations. He showed them again and again that despite the obstacles they faced, he had confidence that they would graduate from college and succeed at whatever they chose to do in life. In return, they loved him unconditionally. This is Dave's personal legacy.

## Comments from Colleagues (in alphabetical order)

### James Casey, Professor, Department of Microbiology and Immunology, Cornell University

Those of you who know artists understand that they see things differently than us. They never introduce themselves as artists, it's apparent that they are. Dave was an artist and actually received some artistic training early in his life. A giveaway was Dave's method when drawing figures during lectures. The nervous circular motion of his hand as he outlined a figure that had clarity, form and completeness suddenly appeared on the board. This unique artistic talent translated in his work as evidenced by the tables and figures that he made for his papers. Photoshop was not available during our time and likely wouldn't have been used by Dave if it were. He ran endless gels until the perfect one emerged that satisfied his artistic sensibility and was scientifically reproducible. One incident, where a contentious plasmid construct wouldn't grow despite numerous attempts and variations, did frustrate us but Dave's reply was, "it's not meant to be." Of course he made successful constructs around the poison sequence and got the answer needed. Dave's unassuming and non-judgmental personality in accepting and kindly dealing with others was punctuated by an impenetrable ego when it came to his own personal science. In the end Dave was given many gifts except for the gift of years.......... "It wasn't meant to be."

### Genoveffa Franchini, Senior Investigator and Chief, Animal Models & Retroviral Vaccines Section, National Cancer Institute/NIH

David was one of the most generous scientist in terms of the time and thoughts he would dedicate to your questions as well as his willingness to provide reagents that may help to address them. His love for the training of young investigators was reflected by his ability to always find time for them. David was reserved, gentle and soft spoken, but you knew you could call him and count on him from help. David had a insatiable scientific curiosity, rigorous and had an unusual ability to always assess the biological importance of everyday experiments. He will be missed by all of us in the field of retrovirology.

### Chou-Zen Giam, Professor of Microbiology and Immunology, Uniformed Services University of the Health Sciences

I have known David since 1986 when his papers in *Science *and the *Journal of Virology *on the comparative study of BLV Tax and HTLV-1 Tax caught my attention. David's work revealed fascinating similarities and differences in the mechanism of action of these two proteins and served as a guide for subsequent works in other labs showing the exquisite specificity of interaction between Tax and the Tax-responsive enhancer elements. Other highlights of David's works include the generation of an infectious molecular clone of HTLV-1 that made reversegenetics possible for HTLV-1; the study of the mechanism of cell-to-cell transmission of HTLV-1; the in-depth analysis of retroviral mRNA splicing and transport; and more recently, the demonstration that the nucleocapsid of HTLV-1 antagonizes the packaging of APOBEC3G into viral particles, to name just a few.

My respect and fondness for David grew over the years after a great deal of interactions in the annual Cold Spring Harbor Retroviruses meetings and the international HTLV meetings. On many occasions, he had alerted me to relevant works from other laboratories and discussed them in the broader context of earlier and present works in the field. As a colleague, David was always generous in providing help and in sharing ideas and reagents. He was a senior statesman in the HTLV/BLV field and served the field in that capacity by organizing workshops to discuss emerging issues and chairing specific sessions. Although David was somewhat of a "private" person, he had a great sense of humor and was a lot of fun to be with when one got to know him. David's untimely death is a great loss to the field and his presence will be sorely missed in the years to come.

### Richard Gontarek, Associate Director, Cancer Research, GlaxoSmithKline Pharmaceuticals

I worked as a Post-doc with Dave from 1994-1996, and although my tenure with him was somewhat brief, he had a profound impact on my development as a scientist and critical thinker. He thrived on discussing raw data hot off the press, but he taught me to always be aware of the larger context so that I could anticipate which experiments to do next. He was someone who could see around scientific corners and he inspired those of us who worked with him to want to do the same. While Dave enjoyed his responsibility to mentor young scientists, he put great effort into learning things from us as well. In his lab he cultivated a deep passion for science, but I will also fondly remember it as a place where labmates could share laughs, listen to classic rock music, and even dance if they wanted to do so. As a scientist, mentor, and friend, Dave was just a great person and will be missed.

### Gisela Heidecker, Staff Scientist, Retrovirus Gene Regulation Section, Drug Resistance Program, National Cancer Institute-Frederick/NIH

Dave Derse was my friend and colleague for 25 years. Most of this time we saw each other daily and worked together closely; he actually was my boss for the last 10 years. He was the best friend, colleague and boss anybody could ask for. He was kind, generous, funny (in a quiet way) and very, very bright. He lived for science, and doing it well was the most important thing to him. "Doing good science" meant to him that you were more interested in getting answers to scientific questions than to beat out the other guy. He was very generous with his time and with reagents he had generated to help the rest of the scientific community. When he still did his own experiments, they were always carefully designed and beautifully executed. Later, when he was responsible for a whole lab, his directions were detailed, when needed, but he also left room for his people to design their own experiments. Under his directions many students and postdocs flourished in the lab and went on to good positions in academia and industry. At this point I still cannot imagine how the lab and I will go on without him. However, we will all try to honor his memory by doing our very best.

### Stephen Hughes, Director, HIV Drug Resistance Program; Chief, Retroviral Replication Laboratory; and Head, Vector Design and Replication Section, National Cancer Institute-Frederick/NIH

I was privileged not only to have Dave Derse as a colleague, but also as a friend and fishing buddy. Most of our trips involved fly fishing for trout in western Maryland. We fished the Casselman in the winter when both the air temperature and the water temperature were 32°F, and a mixture of sleet and rain fell from a leaden sky. We fished Town Creek in the spring when there was the scent of new life in the air and the first hints of green appeared on the ends of the branches above the stream. We fished the Gunpowder in the summer when walking down from the parking lot to the stream in the canyon was like walking from a city sidewalk into an air-conditioned building, and the Savage in the fall when the stream ran gin clear and the fish seemed to be magically suspended above the rocks under a canopy of red and gold. The gift of those days was not the fish we caught, but the chance to be out in some of the most beautiful places on the east coast. I had the best of it, because he was there. In all the years I knew him, I never heard Dave say a negative word about another person, or complain about anything, even when we were, quite literally, wading in ice water. He was one of the kindest and gentlest people I ever knew. His personality illuminated his life and had a profound and positive influence on all who knew him. In the deepest and most original sense, he was a good man.

### Kuan-Teh Jeang, Senior Investigator and Chief, Molecular Virology Section, National Institute of Allergy and Infectious Diseases/NIH

When I heard about David's passing, I was devastated. David's death followed closely on the heels of the passing of three other notable HTLV-1 researchers, Ralph Grassmann [5], John Brady [6], and Bill Harrington [7]. David, like the others, left us all too early. We will remember David as a wonderfully accomplished virologist who made many important contributions, too numerous to list all of them. I do, however, want to mention one of David's papers which has influenced and continues to guide our research [8]. David's construction of an infectious molecular clone for HTLV-1 was a remarkable breakthrough; and even just a few days before David departed, he was emailing my postdoctoral fellow giving us pointers on how to use this HTLV-1 clone. Another point, our most missed friends are ones who do great science and are also outstanding citizens of our retrovirology community. I shall remember David for his services to our community. David was a member of our *Retrovirology *editorial board. He and I also served together on the Norman Salzman Memorial Symposium Committee. A couple of years ago, when I was asked to head the NIH Virology Interest Group (VIG), David was gracious in volunteering to be a member of the VIG advisory board. "Hey, David, say hello to Ralph, John and Bill for me. You guys have some fun up there; don't forget us; we certainly won't forget you.".

### Michael D. Lairmore, Professor and Chair, Department of Veterinary Biosciences; Associate Director for Basic Sciences, Comprehensive Cancer Center, The Ohio State University

David was an outstanding scientist and even a better person. I felt I could trust his results and respected his opinion. He was thorough scientist who asked fundamental questions and tried to use the most current methods to address. Above all else he was curious and his inquisitive nature benefited all that knew him and allowed him to contribute important findings to the retrovirology field. We will miss him dearly.

### Maureen Shuh, Associate Professor, Division of Basic Pharmaceutical Sciences, College of Pharmacy, Xavier University of Louisiana

I was one of the fortunate scientists who worked for Dave as a post-doctoral fellow in his laboratory in 1996-2000. From the time I joined his laboratory, we spent the next 13 years, right up to a few weeks before he became ill, giving each other grief about everything as well as discussing science and politics. Dave was very passionate about science, and his enthusiasm for work was contagious to members of his laboratory. He taught me to be a thorough, careful, thoughtful, and unselfish scientist, but most important, he conveyed the same characteristics in how he treated me as individual. Dave made several important contributions to the field, and at the same time, he never sought accolades for himself. He taught me not only the science but also many aspects of life so that I could be a better person. He watched out for me until the very end of his life. Dave remains the most genuine and kind individual I know. I cherish the years that I worked with him, and I will always miss him.

### Luc Willems, Cellular and Molecular Biology, Agro-Bio Tech (FUSAG), Gembloux, Belgium and Interdisciplinary Cluster for Applied Genoproteomics (GIGA), University of Liège (ULg), Belgium

I had the opportunity to meet Dave as a visiting post-doc in 1990. What I first remember from him is his kindness. He was really a very pleasant guy. He is also one of the best scientists I met in my career. He was extremely cautious and rigorous in his work. He performed essential breakthroughs in the BLV/HTLV field such as Tax-induced transcriptional activation, Rex post-transcriptional regulation and cell-free infection by HTLV-1.
